# Pre-treatment 18F-FDG-PET/CT parameters as biomarkers for progression free survival, best overall response and overall survival in metastatic melanoma patients undergoing first-line immunotherapy

**DOI:** 10.1371/journal.pone.0296253

**Published:** 2024-01-05

**Authors:** Felix Peisen, Annika Gerken, Isabel Dahm, Konstantin Nikolaou, Thomas Eigentler, Teresa Amaral, Jan H. Moltz, Ahmed E. Othman, Sergios Gatidis

**Affiliations:** 1 Department of Diagnostic and Interventional Radiology, Eberhard Karls University, Tuebingen University Hospital, Tuebingen, Germany; 2 Fraunhofer MEVIS, Bremen, Germany; 3 Image-guided and Functionally Instructed Tumor Therapies (iFIT), The Cluster of Excellence (EXC 2180), Tuebingen, Germany; 4 Center of Dermato-Oncology, Department of Dermatology, Eberhard Karls University, Tuebingen University Hospital, Tuebingen, Germany; 5 Department of Dermatology, Venereology and Allergology, Charité – Universitätsmedizin Berlin, Corporate Member of Freie Universität Berlin and Humbolt-Universität zu Berlin, Berlin, Germany; 6 Institute of Neuroradiology, Johannes Gutenberg University Hospital Mainz, Mainz, Germany; 7 Max Planck Institute for Intelligent Systems, Tuebingen, Germany; Università degli Studi di Brescia: Universita degli Studi di Brescia, ITALY

## Abstract

**Background:**

Checkpoint inhibitors have drastically improved the therapy of patients with advanced melanoma. 18F-FDG-PET/CT parameters might act as biomarkers for response and survival and thus can identify patients that do not benefit from immunotherapy. However, little literature exists on the association of baseline 18F-FDG-PET/CT parameters with progression free survival (PFS), best overall response (BOR), and overall survival (OS).

**Materials and methods:**

Using a whole tumor volume segmentation approach, we investigated in a retrospective registry study (n = 50) whether pre-treatment 18F-FDG-PET/CT parameters of three subgroups (tumor burden, tumor glucose uptake and non-tumoral hematopoietic tissue metabolism), can act as biomarkers for the primary endpoints PFS and BOR as well as for the secondary endpoint OS.

**Results:**

Compared to the sole use of clinical parameters, baseline 18F-FDG-PET/CT parameters did not significantly improve a Cox proportional-hazard model for PFS (C-index/AIC: 0.70/225.17 and 0.68/223.54, respectively; p = 0.14). A binomial logistic regression analysis for BOR was not statistically significant (χ^2^(15) = 16.44, p = 0.35), with a low amount of explained variance (Nagelkerke’s R^2^ = 0.38). Mean FDG uptake of the spleen contributed significantly to a Cox proportional-hazard model for OS (HR 3.55, p = 0.04).

**Conclusions:**

The present study could not confirm the capability of the pre-treatment 18F-FDG-PET/CT parameters tumor burden, tumor glucose uptake and non-tumoral hematopoietic tissue metabolism to act as biomarkers for PFS and BOR in metastatic melanoma patients receiving first-line immunotherapy. The documented potential of 18F-FDG uptake by immune-mediating tissues such as the spleen to act as a biomarker for OS has been reproduced.

## Introduction

Therapy of patients with advanced melanoma has seen drastic improvements over the last years since the introduction of the checkpoint inhibitors ipilimumab (targeting the cytotoxic T-lymphocyte associated protein 4 (CTLA-4)) and nivolumab / pembrolizumab (targeting the programmed death-1 (PD-1) receptor) and their combination. Effective immunotherapies are now available that enable treatment regardless of the mutation status [1]. The application of immunotherapeutic drugs has contributed to a significant improvement of patients’ overall survival (OS) and progression free survival (PFS) [2–4].

Unfortunately, about fifty percent of the treated patients present with primary resistance or can develop secondary resistance whilst under immunotherapy. To meet this challenge, clinical markers such as lactate dehydrogenase (LDH) [5, 6] as well as experimental biomarkers are used to identify patients that potentially do not profit from a therapy with checkpoint inhibitors. Standard metric computed tomography (CT) parameters such as tumor size, whole body tumor volume as well as experimental radiomic parameters have been reported in some studies to potentially predict endpoints such as OS, PFS and response after three months [7–12]. On the other hand, there are studies that found no significant prediction capacity of CT parameters in baseline CTs [13]. Currently, no experimental CT biomarker is widely accepted for routine clinical use [14].

Flourine-18 fluorodeoxyglucose positron emission tomography/computed tomography (18F-FDG-PET/CT) is an established method of choice and a cornerstone in the management of patients with metastatic melanoma treated with immune checkpoint inhibitor therapy [15]. It remains an invaluable modality in the response evaluation and monitoring for toxicity of immunotherapies [16]. Based on the findings of recent publications, several 18F-FDG-PET/CT parameters might act as potential biomarkers to predict response and survival in melanoma patients treated with immunotherapy. These parameters can be separated in three groups: tumor burden, tumor glucose uptake and non-tumoral hematopoietic tissue metabolism [17]. Whole-body metabolic tumor volume from 18F-FDG-PET/CT scans acquired approximately three months following initiation of immunotherapy was found to be a strong prognostic indicator of OS in melanoma patients [18]. A pilot study confirmed that the overall survival in patients with unresectable metastatic melanoma undergoing systemic treatment correlated with high tumor load, metastases in certain organ regions, and at least one metastasis with a high diameter or poor metabolism [19]. One study used biomarkers extracted from baseline 18F-FDG-PET/CT before initiation of anti-PD-1 treatment. Total metabolic tumor volume (MTV) correlated with shorter OS and served to define three risk categories [20]. A recent meta-analysis underlined the value of baseline SUVmax, MTV, and total lesion glycolysis (TLG) as promising predictors of the final response to immunotherapy [15]. To our knowledge, only three studies investigated the significance of pre-treatment non-tumoral hematopoietic tissue metabolism (target-to-background ratio, bone marrow-to-liver SUVmax ratio (BLR), SUVmean spleen, and spleen-to-liver SUVmax ratio (SLR)) from baseline 18F-FDG-PET/CT [17, 21, 22]. Summing up, 18F-FDG-PET/CT parameters have the potential to successfully predict response and survival of melanoma patients treated with immunotherapies. However, follow-up examinations are often required to document parameters such as tumor size reduction, the occurrence of new metastases or delta parameters [16, 18, 23]. Little literature exists on the role baseline 18F-FDG-PET/CT and non-tumoral hematopoietic tissue metabolism biomarkers [17].

In a retrospective registry study, using a quantitative segmentation-based approach, we investigated whether pre-treatment 18F-FDG-PET/CT parameters of the three subgroups (tumor burden, tumor glucose uptake and non-tumoral hematopoietic tissue metabolism), based on the segmentation of all metastases in the whole body can act as biomarkers for the primary endpoints progression free survival and best overall response and the secondary endpoint overall survival.

## Material and methods

### Patient selection and workflow overview

**Fig 1:**
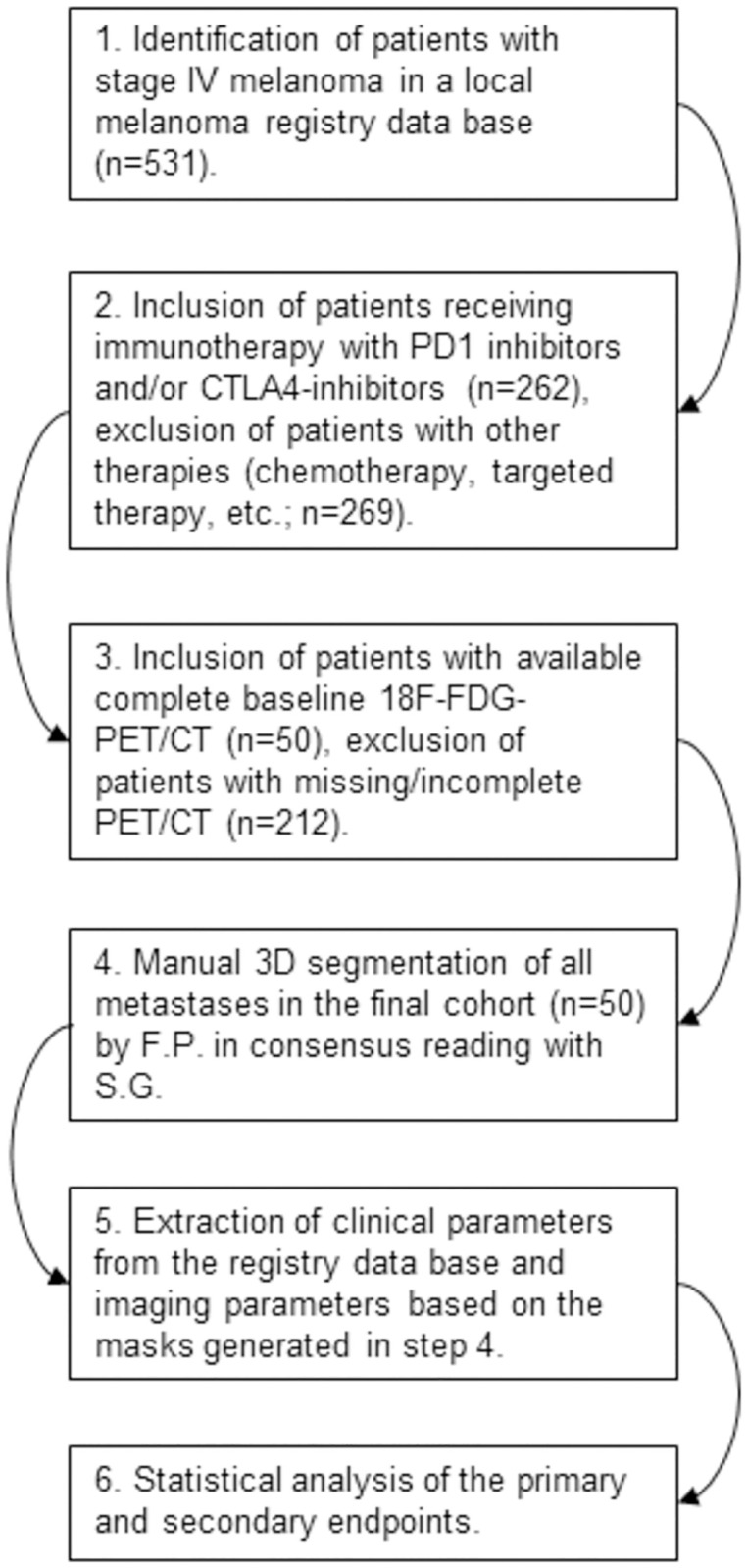
Illustration of the inclusion/exclusion process and study workflow. Abbreviations: 18F-FDG-PET/CT, Flourine-18 fluorodeoxyglucose positron emission tomography/computed tomography; CTLA4, cytotoxic T-lymphocyte-associated protein 4; PD1, programmed death-1.

Patients diagnosed with stage-IV melanoma between January 2015 and December 2018 (AJCC 7^th^ and 8^th^ edition, respectively) were retrospectively identified in a local melanoma registry. All patients were first-line treated with nivolumab, pembrolizumab or ipilimumab mono (n = 36), or with a combination of nivolumab and ipilimumab (n = 14), at the local department for dermatology, according to current guidelines. The study was conducted according to the guidelines of the Declaration of Helsinki and approved by the Institutional Ethics Committee of the Medical Faculty Eberhard-Karls-University Tuebingen (protocol code 092/2019BO2). Inclusion criteria were stage-IV melanoma, first-line treatment with a PD-1 checkpoint inhibitor, a CTLA-4 checkpoint inhibitor, or combination of both, available baseline 18F-FDG-PET/CT scans prior to treatment initiation, available demographic data, and clinical metadata. Exclusion criteria were absence of 18F-FDG-PET/CT baseline imaging, prior treatment with immunotherapy, first-line therapy with targeted therapy or other non-immunotherapies and no visible metastasis on 18F-FDG-PET/CT imaging. For the selected final cohort of 50 patients, all metastatic lesions were manually 3D-segmented in the baseline 18F-FDG-PET/CT images by F.P. (5 years’ experience in oncologic imaging). In the case of unclear lesions, review with S.G. and consensus reading was carried out. The following parameters were extracted per patient: age, gender, baseline S100 serum values [24], baseline LDH values, NRAS (neuroblastoma ras viral oncogene homolog) mutation status [25], BRAF (v-Raf murine sarcoma viral oncogene homolog B1) mutation status [25], Kit (a member of class III transmembrane receptor tyrosine kinases) mutation status [26], total number of lesions, metabolic tumor volume, mean standardized uptake value (SUV) of all tumor lesions, mean SUV spine, mean SUV spleen and mean SUV liver. For an illustration of the inclusion process and workflow, see Fig 1.

### PET/CT imaging

At baseline (prior to treatment initiation), all patients underwent a clinically indicated 18F-FDG-PET/CT scan (Biograph mCT; Siemens Healthcare GmbH, Erlangen, Germany). Patients fasted for at least six hours prior to the injection of 18F-FDG (mean dose: 311.71 +/- 12.72 MBq). The uptake time for the 18F-FDG-PET/CT was 60 min. During PET/CT, a diagnostic contrast-enhanced CT (Ultravist 370; Bayer Healthcare) of the whole body (head to thighs, portal venous phase, expiratory breath-hold) was acquired. An additional thorax scan (inspiratory breath-hold) for the detection of lung metastases was performed. CT acquisition and reconstruction parameters were as follows: 120–140 kV; reference dose: 200 mAs; pitch: 0.7; slice collimation: 128 × 0.6; gantry rotation time: 0.5 s; iterative reconstruction (Safire^®^, Siemens Healthcare GmbH, Erlangen, Germany) kernel B70 or I31f; slice thickness: 3 mm; increment: 2.5 mm; image resolution: 0.9 × 0.9 × 3 mm^3^; matrix size: 512 × 512× 340–1000 (depending on the patient’s size). Whole-body PET scans were performed with a usual scan time per bed position of 2 min. The PET reconstruction parameters were: 3D OP-OSEM time-of-flight reconstruction; 2 mm Gaussian filter; matrix size: 400 × 400. For the attenuation correction, the whole-body CT scan was used.

### Lesion segmentation and measuring imaging biomarkers

All tumor lesions were manually segmented on morphologic information from CT using dedicated software (SATORI, Fraunhofer MEVIS, Bremen, Germany) and subsequently transferred to the PET volume. In case of misalignment, transferred masks were manually corrected using rigid translation. Finally, a 40% SUVmax isocontour threshold was applied to obtain the final segmentation volume for extraction of quantitative features. The resulting masks were used to extract the following parameters: total number of lesions, metabolic tumor volume and mean standardized uptake value of all lesions. Mean SUV spine was computed by drawing a volume-of-interest (VOI) (1 cm radius) in the center of the lumbar vertebral bodies L1 to L4, excluding tumor, metastasis and vertebrae with severe osteoarthritis, fractures or hemangiomas and subsequent averaging of the values. Mean SUV spleen and mean SUV liver were computed by drawing a VOI (2 cm radius) in the spleen and liver respectively, excluding tumor, metastasis, or other lesions. SUV was calculated in a pixel as radioactivity / (injected dose/body weight). Total metabolic tumor volume was calculated as the sum of all tumor-associated voxels (Syngo.via software, Siemens Healthineers GmbH, Erlangen, Germany).

### Endpoints and response evaluation criteria

The primary endpoint analysis tested the capacity of pre-treatment 18F-FDG-PET/CT imaging to predict progression-free survival and best overall response. PFS was defined as the time from the start of immunotherapy to the date of progression or death under first-line immunotherapy. Subjects without progress or death were censored at the date of last follow up. Best overall response (BOR) was dichotomized: Patients who achieved a partial or complete response at any time during the treatment with first-line immunotherapy were defined as responders. Patients who achieved stable disease or progression were classified as non-responders. Response information was extracted from the registry data base, where it was evaluated using the contrast-enhanced follow up CT-scans according to RECIST 1.1 [27], as recommended by the RECIST working group [28]. It was not possible to retrospectively add a response evaluation according to iRECIST, as we did not have access to all follow up scans to check for iRECIST specific endpoints such as confirmed progressive disease after unconfirmed progressive disease (iUPD) [28]. For the same reason a response evaluation with PET criteria (PERCIST) was not possible [29].

The secondary endpoint analysis tested significant pre-treatment 18F-FDG-PET/CT parameters (identified in the primary endpoint analysis for the prediction of PFS and BOR and extracted from literature) for the prediction of overall survival. OS was defined as the time in months from first infusion of immunotherapy to the date of death. Living patients were censored at the time of the last clinical follow up.

### Statistics

To identify potential predictive biomarkers for PFS and OS, Cox proportional-hazard models using Jupyter Notebook were carried out. The likelihood ratio (LR) test was used to compare the C-indices of models. This test requires nested models, i.e., the parameters in one model must be a subset of the parameters of the other model. To identify potential predictive biomarkers for BOR, binominal logistic regression using SPSS 25.0 (IBM) was carried out using forward selection. OS was calculated using the Kaplan Meier method and compared using log rank tests using SPSS 25.0 (IBM). Level of significance was set to p < 0.05.

## Results

### Patient characteristics

A total of 50 patients with stage IV melanoma were included. Most patients in the cohort were male (70%), with a mean age of 66 years. All patients received first-line immunotherapies (ipilimumab, nivolumab, pembrolizumab or nivolumab/ipilimumab). Median follow-up was 34.42 months (range: 0.6–81). 46% of the cohort had complete or partial response. Mean progression free survival for patients with progressive disease was 181 days and 18 patients (36%) had died during follow up. Detailed patient characteristics are summarized in Table 1.

**Table 1:** Patients characteristics.

Variable	Mean [range], n (%)
**Clinical characteristics**	
Age stage IV (y)	66 [33–89]
Age Group	
<60y	19 (38)
60y-75y	16 (32)
>75y	15 (30)
Gender	
m	35 (70)
f	15 (30)
BRAF mutation	16 (32)
Kit mutation	0 (0)
NRAS mutation	6 (12)
LDH baseline (U/l)	233.68 [137–443]
LDH elevation (>250 U/l)	13 (26)
S100 baseline (μg/l)	0.46 [0.03–9.47]
S100 elevation (> 0.1 μg/l)	25 (50)
**Treatment**	
Ipilimumab (CTLA-4)	6 (12)
Pembrolizumab (PD-1)	23 (46)
Nivolumab (PD-1)	7 (14)
Nivolumab + Ipilimumab	14 (28)
**Response**	
Response after 3 months (RECIST 1.1)	
CR	4 (8)
PR	16 (32)
SD	8 (16)
PD	19 (38)
Missing	3 (6)
Best overall response (RECIST 1.1)	
CR	15 (30)
PR	8 (16)
SD	6 (12)
PD	20 (40)
CR+PR	23 (46)
SD+PD	26 (52)
Missing	1 (2)
**Survival**	
PFS (d) for PD	180.96 [5–1094]
Duration PFS	
< 1 year	28 (56)
> 1 year	22 (44)
OS (m)	51.00 [2–81]

Abbreviations: BRAF, v-Raf murine sarcoma viral oncogene homolog B1; CR, complete response; CTLA-4, cytotoxic T-lymphocyte-associated protein 4; LDH, lactate dehydrogenase; NRAS, neuroblastoma ras viral oncogene homolog; OS, overall survival; PD, progressive disease; PD-1, programmed death 1; PFS, progression free survival, PR, partial response; RECIST, Response Evaluation Criteria In Solid Tumors; SD, stable disease;

### Cox proportional-hazard model for progression free survival

A Cox proportional-hazard model was performed to determine the associations of the variables listed in Table 2 with progression free survival. No variable was independent statistically significant. The concordance index and the Akaike information criterion (AIC) for the model containing all parameters (clinical + PET/CT) were 0.70 and 225.17, respectively. The concordance index and the Akaike information criterion for the model containing only clinical parameters were 0.68 and 223.54, respectively. Baseline PET/CT imaging parameters (number of lesions, metabolic tumor volume, mean tumor uptake, SUVmean spine, SUVmean spleen, SUVmean liver) did not contribute to an improvement of the model. The difference of the C-indices of the two models was not statistically significant (p = 0.14, LR test). For detailed values, see Table 2.

**Table 2:** Results of the Cox proportional-hazard model for progression free survival.

**Model with PET/CT parameters**			
**Variable**	**Hazard Ratio**	**95% CI**	**p**
Age	0.98	0.94–1.02	0.32
Gender	2.26	0.82–6.22	0.11
Baseline LDH	1.00	0.99–1.01	0.38
Baseline S100	0.81	0.17–0.56	0.28
NRAS mutation	2.21	0.49–9.96	0.30
BRAF mutation	0.93	0.35–2.48	0.88
Number of lesions	1.00	0.99–1.03	0.34
Metabolic tumor volume	1.00	0.00–1.00	0.26
Mean tumor uptake	0.98	0.13–0.84	0.78
Mean uptake spine	0.23	0.05–1.14	0.07
Mean uptake spleen	1.06	0.46–2.42	0.89
Mean uptake liver	0.84	0.21–3.41	0.80
Concordance index	0.70		
AIC	225.17		
**Model without PET/CT parameters**			
**Variable**	**Hazard Ratio**	**95% CI**	**p**
Age	0.99	0.96–1.03	0.69
Gender	2.00	0.89–4.51	0.09
Baseline LDH	1.00	0.99–1.01	0.57
Baseline S100	0.83	0.58–1.19	0.32
NRAS mutation	1.86	0.41–8.50	0.42
BRAF mutation	0.87	0.40–1.90	0.72
Concordance index	0.68		
AIC	223.54		

Abbreviations: AIC, Akaike information criterion; BRAF, v-Raf murine sarcoma viral oncogene homolog B1; LDH, lactate dehydrogenase; NRAS, neuroblastoma ras viral oncogene homolog

### Binominal logistic regression for binary best overall response

**Fig 2:**
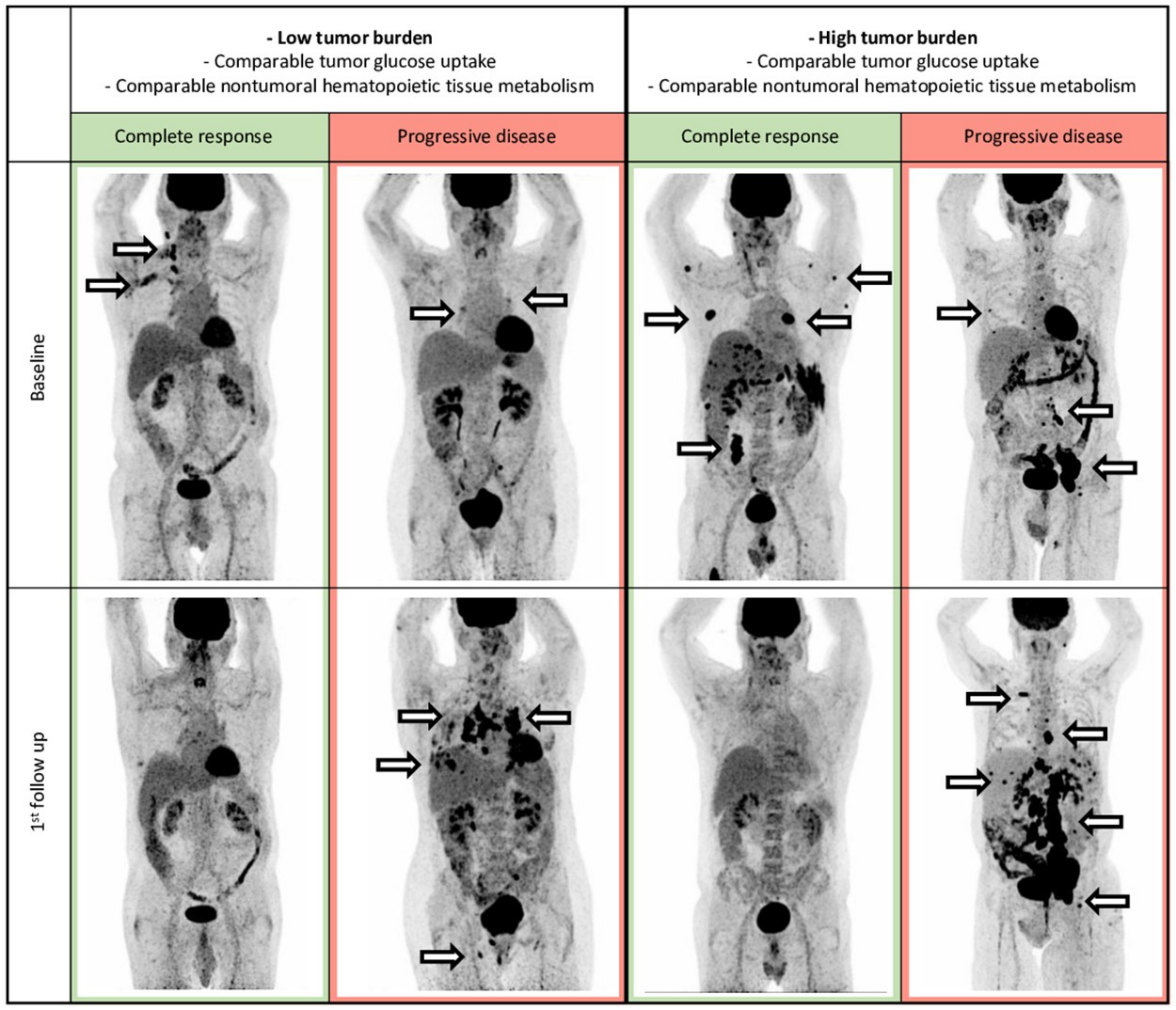
Maximum intensity projection baseline and 1st follow up PET images. Examples of responders (1st and 3rd column) and non-responders (2nd and 4th column) grouped by low tumor burden vs high tumor burden with comparable tumor glucose uptake and non-tumoral hematopoietic tissue metabolism. Arrows exemplary mark tumor manifestations with elevated FDG uptake.

A binomial logistic regression analysis was performed to determine the association of the variables listed in Table 3 with binary best overall response. The binomial logistic regression model was not statistically significant (χ^2^(15) = 16.44, p = 0.35), resulting in a low amount of explained variance, as shown by Nagelkerke’s R^2^ = 0.38 [30]. Of the twelve variables that entered the regression model, none contributed independent significantly to the model. All model coefficients and odds can be found in Table 3. Fig 2 provides exemplary cases, illustrating the heterogeneity of tumor burden within the responder and non-responder group, respectively, with comparable tumor glucose uptake and non-tumoral hematopoietic tissue metabolism.

**Table 3:** Results of the binominal logistic regression for binary best overall response.

	B	SE	Wald	p	Odds Ratio
Constant	5.39	4.10	1.73	0.19	219.25
Age	-0.05	0.04	1.57	0.21	0.95
Gender	1,16	0.98	1.42	0.23	3.20
Baseline LDH	0.01	0.01	0.01	1.00	1.00
Baseline S100	-0.18	0.26	0.49	0.49	0.84
NRAS mutation	-1.60	2.08	0.59	0.44	0.20
BRAF mutation	-0.70	0.99	0.49	0.48	0.50
Number of lesions	0.03	0.02	1.42	0.23	1.03
Metabolic tumor volume	0.01	0.01	0.15	0.70	1.00
Mean tumor uptake	-0.02	0.10	0.03	0.86	0.98
Mean uptake spine	-2.35	1.58	2.22	0.14	0.10
Mean uptake spleen	0.54	0.99	0.29	0.59	1.71
Mean uptake liver	-0.46	1.47	0.10	0.75	0.63

*p<0.05; **p<0.01; ***p<0.001

Abbreviations: BRAF, v-Raf murine sarcoma viral oncogene homolog B1; LDH, lactate dehydrogenase; NRAS, neuroblastoma ras viral oncogene homolog

### Cox proportional-hazard model for overall survival

A Cox proportional-hazard model was performed to determine the association of the variables listed in Table 4 with overall survival. The only parameter contributing significantly to the model was the mean FDG uptake of the spleen (HR 3.55, p = 0.04). Subsequently, a Kaplan-Meier estimator with a log-rank test was performed to assess whether patients could be grouped into a low- and high-risk group, clustered by spleen uptake below and above median (3.84). Results showed that survival distributions of the two groups did not differ significantly (χ^2^(1) = 0.62, *p* = 0.43). OS was 54.00 months (95% CI: 42.70–65.32) and 49.00 months (95% CI: 42.01–55.93) respectively.

**Table 4:** Results of the Cox proportional-hazard model for prediction of overall survival.

Variable	Hazard Ratio	95% CI	p
Age	1.03	0.99–1.08	0.15
Gender	1.00	0.33–2.99	0.99
Baseline LDH	1.00	0.99–1.01	0.76
Baseline S100	1.27	0.96–1.67	0.09
NRAS mutation	0.64	0.34–1.17	0.15
BRAF mutation	0.72	0.21–2.45	0.60
Number of lesions	1.00	0.97–1.04	0.73
Metabolic tumor volume	1.00	1.00–1.00	0.70
Mean tumor uptake	0.96	0.79–1.18	0.72
Mean uptake spine	2.23	0.36–13.74	0.39
Mean uptake spleen	3.55	1.10–11.56	**0.04**
Mean uptake liver	0.23	0.04–1.36	0.10

Abbreviations: BRAF, v-Raf murine sarcoma viral oncogene homolog B1; LDH, lactate dehydrogenase; NRAS, neuroblastoma ras viral oncogene homolog

### Kaplan-Meier estimator for OS grouped by binary best overall response (CR+PR/SD+PD)

**Fig 3:**
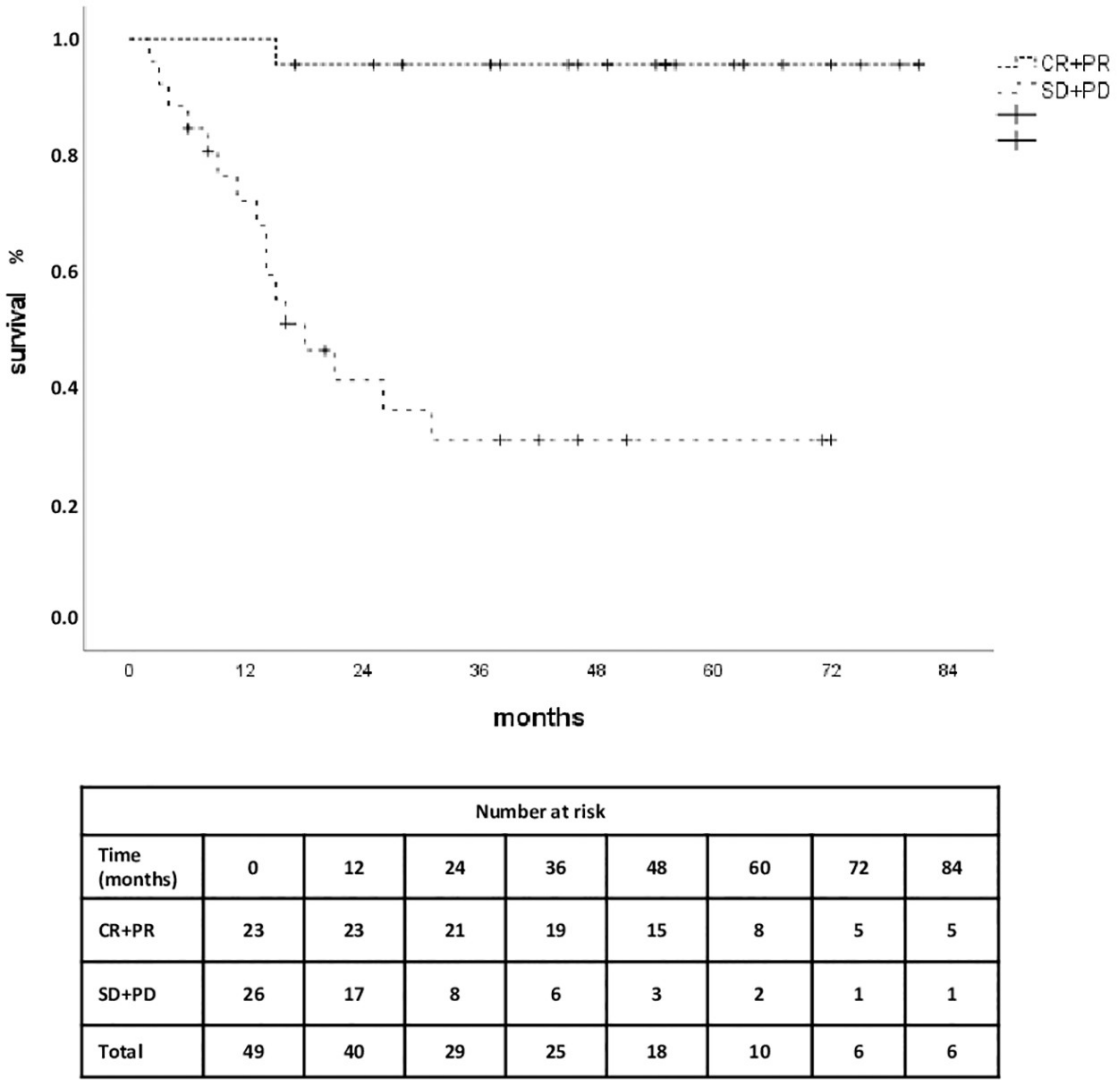
Kaplan-Meier estimator for overall survival grouped by binary best overall response during first-line immunotherapy. OS for CR/PR (RECIST 1.1) = 78.13 months (95% CI: 72.63–83.63); OS for SD/PD (RECIST 1.1) = 31.85 months (95% CI: 20.43–43.28 months). Log-rank test: χ^2^(2) = 20.31, p < 0.001. Abbreviations: CI, confidence interval; CR, complete response; PD, progressive disease, PR, partial response; SD, stable disease; OS, overall survival.

As none of the baseline imaging parameters was significantly independent in the models for PFS and BOR and only one parameter in the model for OS, we carried out a Kaplan-Meier analysis for OS grouped by binary best overall response to evaluate if our sample was behaving as expected and could act as a representative cohort (compare Fig 3). Best overall response is a documented [31] parameter for the prediction of OS; however, we did not include it in the primary analysis as it is not a baseline parameter. OS for patients with CR or PR according to RECIST 1.1 criteria was longer (78.13 months; 95% CI: 72.63–83.63) than for patients with SD or PD (31.85 months; 95% CI: 20.43–43.28 months). Results from a subsequent log-rank test showed that survival distributions of the two groups did differ significantly (χ^2^(2) = 20.31, p < 0.001).

## Discussion

In a retrospective registry study using a quantitative whole tumor volume segmentation approach, we investigated whether baseline 18F-FDG-PET/CT parameters are associated with progression free survival, best overall response and overall survival in a stage IV melanoma cohort undergoing first-line treatment with immunotherapy. We investigated parameters from three subgroups: tumor burden (number of lesions, MTV), tumor glucose uptake (mean uptake of all lesions) and non-tumoral hematopoietic tissue metabolism (mean uptake spine, mean uptake spleen). Our sample consisted mainly of male patients (70%) older than 60 years, with a typical distribution of response after three months (8% CR, 38% PR, 32% SD, 16% PD) and best overall response to first-line immunotherapy (30% CR, 16% PR, 12% SD, 40% PD) according to RECIST 1.1 criteria [27]. Binary distribution of responder vs. non-responder was almost equal (46% CR+PR vs 52% SD+PD).

Mean progression free survival in our cohort was 181 days, correlating with results published by Weide et al. [32] and Awada et al. [33]. A Cox proportional-hazard model for progression free survival showed that independently, none of the investigated parameters contributed significantly to the model. The inclusion of baseline PET/CT parameters did not lead to an improvement of the model. Based on the results published by several authors [17, 20, 21, 33, 34] we would have expected the baseline 18F-FDG-PET/CT parameters mean spine FDG uptake and mean spleen FDG uptake (as surrogate parameters for hematopoietic tissue metabolism [17]) as well as MTV (as a surrogate parameter for tumor burden [17, 20, 33, 34]) to serve as biomarkers. However, none of the investigated baseline PET/CT imaging parameters revealed a predictive capacity. Three explanations for these contrary results must be discussed. Firstly, differences in the samples might have influenced the results: Whilst the mentioned authors included only patients treated with anit-PD-1 monotherapy, our sample included patients treated with ipilimumab, nivolumab or pembrolizumab mono, or with a combination of nivolumab and ipilimumab. Awada et al. and Seban et al. included patients with prior immunotherapy treatment [17, 33]. Ito et al. included 20 patients (14%) with mucosal and 9 patients (6%) with uveal melanoma, two histological subtypes with very different behavior compared to cutaneous melanoma [34]. Secondly, our primary endpoints partly differed compared to the before mentioned studies (PFS vs OS). However, as both endpoints are strongly connected, we assumed a transferability. Thirdly, Seban et al. hypothesized that including bone marrow and spleen FDG uptake measurements, as surrogate parameters for medullary and extra-medullary haematopoiesis, provides a source of complementary prognostic information, as a pro-inflammatory immune response is elicited in cancer associated lymphoid tissues [17]. They chose to dichotomize the PET parameters and normalize the non-tumoral hematopoietic tissue metabolism parameters (spleen to liver ratio and bone marrow to liver ratio), similar to Nakamoto et al. [21]. We decided to primarily investigate the raw data (mean spleen uptake, mean spine uptake, mean tumor uptake, MTV). However, additional analysis of dichotomized and normalized spleen to liver and spine to liver ratios, dichotomized SUVmean spleen, SUVmean spine and tumor SUVmean as well as dichotomized MTV, using the median SUV as a separator, did not show significant results of baseline imaging parameters as well for the endpoints PFS and BOR (data reported in supporting files).

In a binary logistic regression model for best overall response, none of the selected baseline 18F-FDG-PET/CT parameters was independently associated with BOR. Fig 2 provides exemplary cases illustrating the heterogeneity of tumor burden within the responder and non-responder group, with comparable tumor glucose uptake and non-tumoral hematopoietic tissue metabolism, respectively. This example graphically highlights the non-predictive distribution of baseline PET/CT parameters in the responder and non-responder groups. Based on the results published by Seban et al. [17], we would have expected MTV, as a surrogate parameter for tumor burden, to serve as a predictor. They reported low tumor burden to correlate with best overall response; however, our results revealed a non-significant odds ratio of 1.00 (p = 0.70) for MTV. A potential explanation might be the inherent heterogeneity of our sample. Whilst Seban et al. investigated a sample of patients treated only with anti-PD-1 receptor antibodies, our sample included patients both treated with ipilimumab, nivolumab or pembrolizumab mono, or with a combination of nivolumab and ipilimumab, according to current guidelines. Moreover, Seban et al. included 18 patients (34%) that received prior treatment with ipilimumab, our sample on the other hand was strictly restricted to patients receiving first-line treatment with immunotherapy. Despite the similar sample size and otherwise similar patient’s characteristics, these differences might explain our deviating results.

Based on the results of the primary endpoint analysis, no explicit parameter entered the secondary endpoint analysis. Instead, we further investigated all parameters included in the analysis for the endpoints PFS and BOR that were derived from publications by other authors investigating pre-treatment parameters [17, 20, 33–35]. Mean FDG uptake of the spleen was the only independent significant variable in a Cox proportional-hazard model, indicating that an elevated FDG uptake of the spleen correlated with a decreased OS. This is in line with results published by Seban et al. [17], Nakamoto et al. [21] and Wong et al. [35], who reported that hematopoietic tissue metabolism could serve as a biomarker for OS. A pathophysiological explanation for the negative correlation might be the connection of hematopoietic tissue-derived cells and tumor progression, neovascularization, and priming of metastasis [21, 36, 37]. Moreover, the complex interplay between local and systemic inflammatory factors causes immunosuppression. This interaction alters the disease course with potential cancer progression and poor outcomes [21, 38]. Wong et al. defined a SLR > 1.1 as predictive for a decreased OS [35]. We could detect a trend towards this benchmark; however, our results must be considered with caution, as only 4% of our cohort had an elevated SLR > 1.1 (see S1 Fig in S1 File). Subsequently, we aimed to define a low-risk and high-risk group for OS, based on a separator by the median SLR (0.78). The results revealed that no statistically significant separation was accomplished; however, again a trend toward lower OS in patients with elevated SLR was reproduced (see S2 Fig in S1 File). To confirm that, despite the mostly negative results of the analysis for PFS, BOR and OS, our cohort can act as a representative sample, we finally carried out a Kaplan-Meier estimator and log rank test for the correlation of dichotomized BOR and OS. Complete and partial response according to RECIST 1.1 strongly predicted a prolonged overall survival, compared to stable disease and progressive disease. This behavior was expected and in line with the results published by Wang et al. [31]. In synopsis with the demographic data, we can therefore assume that our cohort can act as a representative and well documented sample of stage IV melanoma patients. Despite the prospective documentation in a registry, the sample remains small. Other studies used sample sizes comparable to the present cohort [17, 35], nevertheless, a larger sample would have been of advantage. Lesion segmentation was carried out by only one radiologist, due to the whole-body segmentation approach. We tried to compensate for this limitation with a consensus reading with a second and well experienced radiologist. Finally, our sample consists of patients treated both with PD-1 and CTLA-4 antibodies mono, and with a combination of both. A possible drawback of this approach is the documented lower rate of responders with Ipilimumab monotherapy, compared to anti-PD1 monotherapy and combined anti-PD-1 and CTLA-4 therapy [39–41] and a subsequent bias in the endpoint analysis. Due to the small sample size, only additional analysis of the anti-PD1 monotherapy sub-cohort was possible.

## Conclusions

Summing up, the present study could not confirm the capability of the pre-treatment 18F-FDG-PET/CT parameters tumor burden, tumor glucose uptake and non-tumoral hematopoietic tissue metabolism to act as biomarkers for progression free survival and best overall response in metastatic melanoma patients receiving first-line immunotherapy. The documented potential of 18 F-FDG uptake by immune-mediating tissues such as the spleen (as well as the non-baseline parameter BOR) to act as biomarkers for overall survival has been reproduced.

## Supporting information

S1 File (DOCX)
