# Supplementary material for: Pre-treatment 18F-FDG-PET/CT parameters as biomarkers for progression free survival, best overall response and overall survival in metastatic melanoma patients undergoing first-line immunotherapy
Source: PLoS One. 2024 Jan 5;19(1):e0296253. doi: 10.1371/journal.pone.0296253 (PMC10769042; doi:10.1371/journal.pone.0296253)
Supplement: S1 File — (DOCX) [file pone.0296253.s001.docx]

**Supporting information**


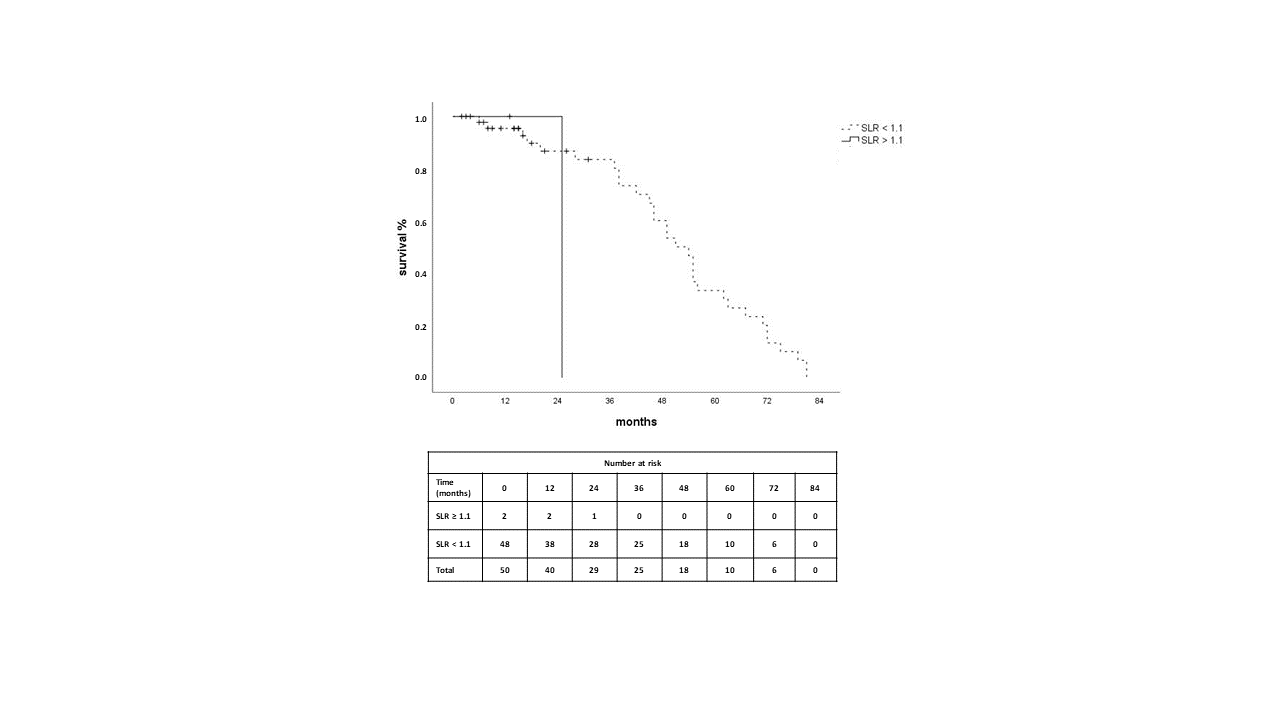


S1 Figure: Kaplan-Meier estimator for OS grouped by SLR ≥ 1.1/< 1.1. Additional log-rank tests revealed that OS in the group with SLR ≥ 1.1 was lower than in the group with SLR < 1.1 (25.00 months (95% CI 25.00-25.00) vs 50.85 months (95% CI 44.05-57.64) respectively). Level of significance was not reached (p = 0.08).


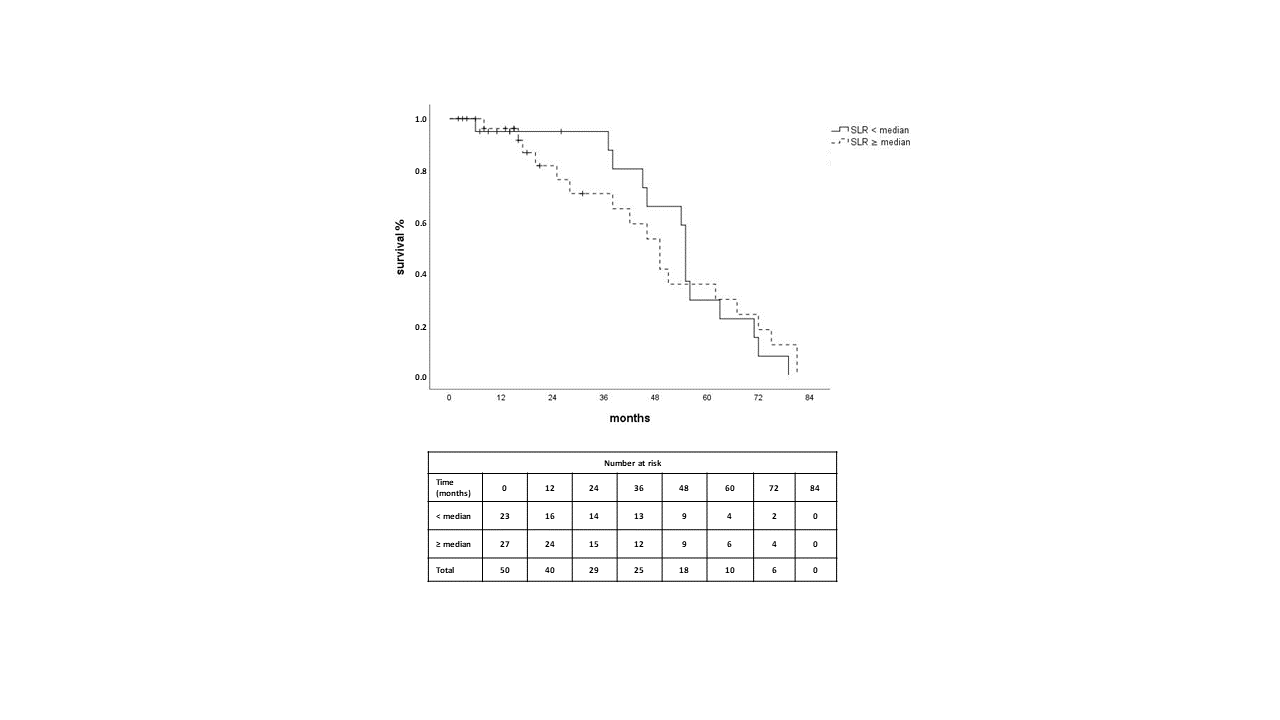


S2 Figure: Kaplan-Meier estimator for OS grouped by SLR ≥ median/< median. Additional log-rank tests revealed that OS in the group with SLR ≥ median was lower than in the group with SLR < median (47.81 months (95% CI 37.64-57.99) vs 53.35 months (95% CI 45.11-61.59) respectively). Level of significance was not reached (p = 0.95).


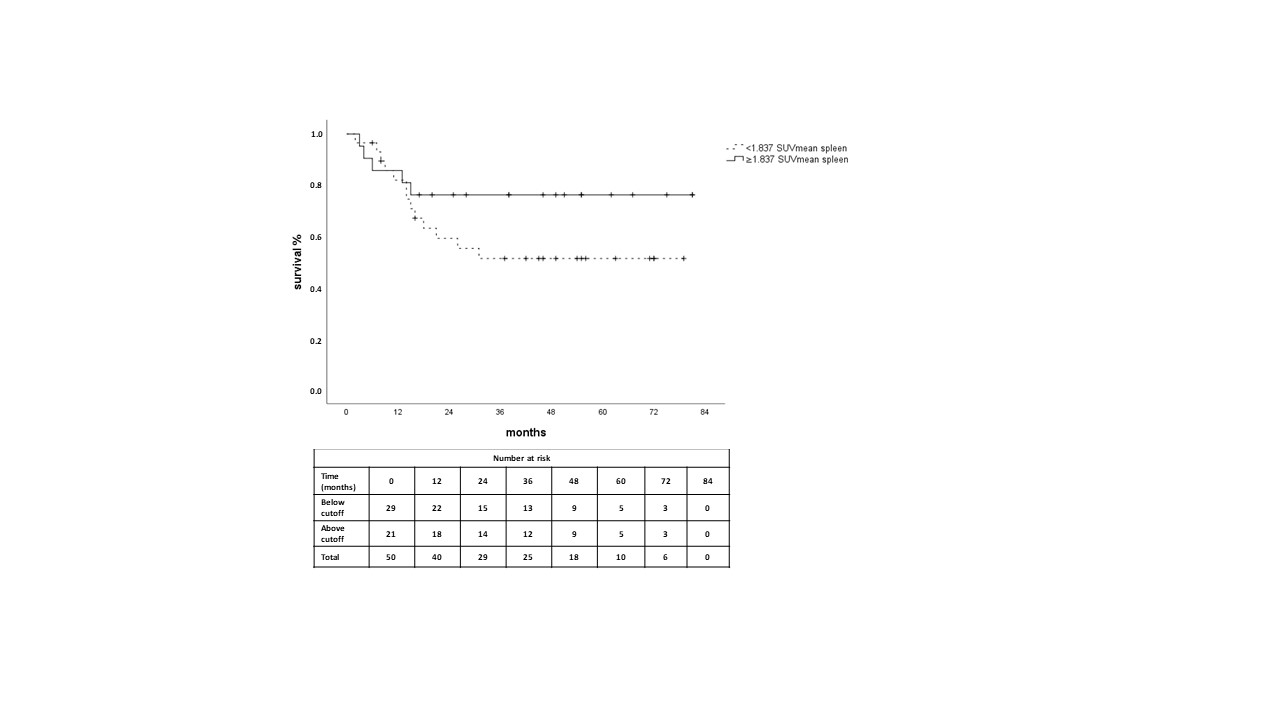


S3 Figure: Kaplan-Meier estimator for OS grouped by SUVmean spleen ≥ 1.837/< 1.837. Additional log-rank test revealed a non-significant difference of the two groups (χ²(1) = 1.92, p = 0.17). OS was 63.67 months (95% CI: 50.37-76.97) and 47.84 months (95% CI: 35.52-60.16)

S1 Table: Results of the Cox proportional-hazard model of additional parameters (dichotomized and normalized spleen to liver and spine to liver ratios, dichotomized SUVmean spleen, SUVmean spine and SUVmean tumor as well as dichotomized MTV, using the median SUV as a separator, SUVmax liver, SUVmax spleen, SUVmax spine and SUVmax tumor) for progression free survival.

| **Variable** | **Hazard Ratio** | **95% CI** | **p** |
| --- | --- | --- | --- |
| Dichotomized and normalized spleen to liver ratio | 3.66 | 0.41-32.35 | 0.24 |
| Dichotomized and normalized spine to liver ratio | 0.25 | 0.04-1.58 | 0.14 |
| Dichotomized SUVmean spleen | 0.83 | 0.13-5.43 | 0.84 |
| Dichotomized SUVmean spine | 2.03 | 0.40-10.22 | 0.39 |
| Dichotomized SUVmean tumor | 0.95 | 0.34-2.69 | 0.92 |
| Dichotomized MTV | 0.59 | 0.21-1.68 | 0.32 |
| SUVmax liver | 0.92 | 0.83-1.03 | 0.42 |
| SUVmax spleen | 1.21 | 1.00-1.47 | 0.05 |
| SUVmax spine | 0.96 | 0.87-1.06 | 0.42 |
| SUVmax tumor | 1.04 | 0.97-1.12 | 0.31 |
| Abbreviations: MTV, metabolic tumor volume; SUV, standardized uptake value | | | |

S2 Table: Results of the binominal logistic regression of additional parameters (dichotomized and normalized spleen to liver and spine to liver ratios, dichotomized SUVmean spleen, SUVmean spine and SUVmean tumor as well as dichotomized MTV, using the median SUV as a separator, SUVmax liver, SUVmax spleen, SUVmax spine and SUVmax tumor) for binary best overall response.

|  | **B** | **SE** | **Wald** | **p** | **Odds Ratio** |
| --- | --- | --- | --- | --- | --- |
| Constant | -0.35 | 0.68 | 0.27 | 0.60 | 0.70 |
| Dichotomized and normalized spleen to liver ratio | -0.09 | 0.86 | 0.10 | 0.92 | 0.92 |
| Dichotomized and normalized spine to liver ratio | -1.30 | 0.92 | 2.0 | 0.16 | 0.27 |
| Dichotomized SUVmean spleen | 0.49 | 0.85 | 0.33 | 0.57 | 1.63 |
| Dichotomized SUVmean spine | 1.52 | 0.88 | 2.99 | 0.84 | 4.57 |
| Dichotomized SUVmean tumor | 0.57 | 0.68 | 0.71 | 0.40 | 1.77 |
| Dichotomized MTV | -0.24 | 0.66 | 0.13 | 0.72 | 0.80 |
| SUVmax liver | -0.04 | 0.06 | 0.59 | 0.62 | 1.04 |
| SUVmax spleen | 0.08 | 0.08 | 0.98 | 0.32 | 1.09 |
| SUVmax spine | 0.03 | 0.07 | 0.25 | 0.62 | 1.04 |
| SUVmax tumor | -0.05 | 0.03 | 2.77 | 0.10 | 0.95 |
| Abbreviations: MTV, metabolic tumor volume; SUV, standardized uptake value | | | | | |

S3 Table: Results of the Cox proportional-hazard model of additional parameters (SUVmax liver, SUVmax spleen, SUVmax spine, SUVmax tumor) for overall survival.

| **Model with PET/CT parameters** |  |  |  |
| --- | --- | --- | --- |
| **Variable** | **Hazard Ratio** | **95% CI** | **p** |
| SUVmax liver | 1.01 | 0.92-1.11 | 0.85 |
| SUVmax spleen | 0.95 | 0-78-1.15 | 0.57 |
| SUVmax spine | 1.05 | 0.97-1.15 | 0.24 |
| SUVmax tumor | 0.97 | 0.93-1.02 | 0.22 |
| Abbreviations: MTV, metabolic tumor volume; SUV, standardized uptake value | | | |

S4 Table: Results of the Cox proportional-hazard model of PET parameters (SUVmean liver, SUVmean spleen, SUVmean spine, SUVmean tumor, SUVmax liver, SUVmax spleen, SUVmax spine, SUVmax tumor) for progression free survival in the subgroup treated with anti-PD1 monotherapy.

| **Model with PET/CT parameters** |  |  |  |
| --- | --- | --- | --- |
| **Variable** | **Hazard Ratio** | **95% CI** | **p** |
| MTV | 1.00 | 1.00-1.00 | 0.45 |
| SUVmean liver | 0.48 | 0.19-1.24 | 0.13 |
| SUVmean spleen | 0.80 | 0.35-1.81 | 0.59 |
| SUVmean spine | 0.33 | 0.10-1.14 | 0.08 |
| SUVmean tumor | 1.23 | 0.87-1.76 | 0.25 |
| SUVmax liver | 0.80 | 0.61-1.06 | 0.12 |
| SUVmax spleen | 1.27 | 0.70-2.31 | 0.43 |
| SUVmax spine | 0.80 | 0.56-1.14 | 0.22 |
| SUVmax tumor | 0.80 | 0.72-1.01 | 0.07 |
| Abbreviations: MTV, metabolic tumor volume; SUV, standardized uptake value | | | |

S5 Table: Results of the binominal logistic regression of PET parameters (SUVmean liver, SUVmean spleen, SUVmean spine, SUVmean tumor, SUVmax liver, SUVmax spleen, SUVmax spine, SUVmax tumor) for binary best overall response in the subgroup treated with anti-PD1 monotherapy.

|  | **B** | **SE** | **Wald** | **p** | **Odds Ratio** |
| --- | --- | --- | --- | --- | --- |
| Constant | 8.37 | 4.32 | 3.75 | 0.05 | 4302.67 |
| MTV | 0.01 | 0.01 | 0.04 | 0.84 | 1.00 |
| SUVmean liver | -3.36 | 2.96 | 1.29 | 0.26 | 0.04 |
| SUVmean spleen | 3.31 | 3.74 | 0.79 | 0.38 | 27.40 |
| SUVmean spine | -1.93 | 2.15 | 0.81 | 0.37 | 0.15 |
| SUVmean tumor | 0.25 | 0.23 | 1,24 | 2.67 | 1.29 |
| SUmax liver | 0.11 | 0.10 | 1.16 | 0.28 | 1.11 |
| SUVmax spleen | -0.38 | 0.31 | 1.44 | 0.23 | 0.69 |
| SUVmax spine | 0.04 | 0.10 | 0.13 | 0.72 | 1.04 |
| SUVmax tumor | -0.20 | 0.11 | 3.10 | 0.08 | 0.82 |
| Abbreviations: MTV, metabolic tumor volume; SUV, standardized uptake value | | | | | |

S6 Table: Results of the Cox proportional-hazard model of PET parameters (SUVmean liver, SUVmean spleen, SUVmean spine, SUVmean tumor, SUVmax liver, SUVmax spleen, SUVmax spine, SUVmax tumor) for overall survival in the subgroup treated with anti-PD1 monotherapy.

| **Model with PET/CT parameters** |  |  |  |
| --- | --- | --- | --- |
| **Variable** | **Hazard Ratio** | **95% CI** | **p** |
| MTV | 1.00 | 1.00-1.00 | 0.76 |
| SUVmean liver | 0.84 | 0.23-2.98 | 0.78 |
| SUVmean spleen | 1.01 | 0.47-2.46 | 0.87 |
| SUVmean spine | 0.80 | 0.19-3.30 | 0.75 |
| SUVmean tumor | 1.01 | 0.69-1.47 | 0.97 |
| SUmax liver | 0.70 | 0.50-0.99 | 0.41 |
| SUVmax spleen | 1.67 | 0.60-4.68 | 0.32 |
| SUVmax spine | 1.14 | 0.86-1.15 | 0.36 |
| SUVmax tumor | 1.01 | 0.82-1.25 | 0.94 |
| Abbreviations: MTV, metabolic tumor volume; SUV, standardized uptake value | | | |
